# A Study on a Low-Cost IMU/Doppler Integrated Velocity Estimation Method Under Insufficient GNSS Observation Conditions

**DOI:** 10.3390/s25247674

**Published:** 2025-12-18

**Authors:** Yinggang Wang, Hongli Zhang, Kemeng Li, Hanghang Xu, Yijin Chen

**Affiliations:** College of Geoscience and Surveying Engineering, China University of Mining and Technology (Beijing), Beijing 100083, China; bqt2200205055@student.cumtb.edu.cn (Y.W.); bqt2100205061@student.cumtb.edu.cn (H.Z.); bqt2200205054@student.cumtb.edu.cn (K.L.); bqt2000205068@student.cumtb.edu.cn (H.X.)

**Keywords:** GNSS/INS integration, Doppler velocity estimation, complex urban environments, robust navigation, low-cost navigation

## Abstract

**Highlights:**

**What are the main findings?**

- A low-cost differential Doppler and IMU attitude-aided velocity estimation method is proposed, which enables full 3D velocity determination even with only two visible satellites.
- A Doppler–IMU integrated velocity model is established based on satellite differential geometry and vehicle motion constraints, and its effectiveness is validated using real vehicle experiments in complex urban environments.

**What are the implications of the main findings?**

- The proposed method significantly enhances the continuity and robustness of navigation systems under weak GNSS conditions, providing a practical solution for field applications.

**Abstract:**

The Global Navigation Satellite System (GNSS)/Inertial Measurement Unit (IMU) Loosely Coupled (LC) integration framework has been widely adopted due to its simple structure, but it relies on complete GNSS position and velocity solutions, and the rapid accumulation of IMU errors can easily lead to navigation failure when fewer than four satellites are visible. In this paper, GNSS Doppler observations are fused with IMU attitude information within an LC framework. An inter-satellite differential Doppler model is introduced, and the velocity obtained from the differential Doppler solution is transformed into the navigation frame using the IMU-derived attitude, enabling three-dimensional velocity estimation in the navigation frame even when only two satellites are available. Analysis of real vehicle data collected by the GREAT team at Wuhan University shows that the Signal-to-Noise Ratio (SNR) and the geometric relationship between the Satellite Difference Vector (SDV) and the Receiver Motion Direction (RMD) are the dominant factors affecting velocity accuracy. A multi-factor threshold screening strategy further indicates that when SNR> 40 and ∣SDV·RMD∣ >0.2, the Root Mean Square (RMS) of the velocity error is approximately 0.3 m/s and the data retention rate exceeds 44%, achieving a good balance between accuracy and availability. The results indicate that, while maintaining a simple system structure, the proposed Doppler–IMU fusion method can significantly enhance velocity robustness and positioning continuity within an LC architecture under weak GNSS conditions (when more than two satellites are visible but standalone GNSS positioning is still unavailable), and is suitable for constructing low-cost, highly reliable integrated navigation systems.

## 1. Introduction

With the continuous advancement of GNSS and Inertial Navigation System (INS) technologies, integrated GNSS/INS navigation has been widely applied in aerospace, autonomous driving, precision agriculture, and other fields. By combining the long-term stability of GNSS with the short-term high accuracy of INS, GNSS/INS fusion systems significantly enhance positioning accuracy and robustness. However, in complex environments such as urban canyons, tunnels, and underground passages, GNSS signals are easily blocked or interfered with, resulting in a significant reduction in the number of visible satellites and severe signal degradation, which negatively affects the continuity and accuracy of positioning [1,2,3,4,5]. Therefore, improving the robustness of GNSS/INS fusion systems under weak GNSS conditions has become a critical research focus [6].

Currently, GNSS/INS integration typically employs two typical architectures: LC and Tightly Coupled (TC). The LC structure is widely used in industrial-grade navigation systems due to its simplicity, ease of implementation, and low computational cost [3,7]. Its basic principle is to use GNSS-derived position and velocity as observation inputs, which are then fused with INS outputs, commonly using the Extended Kalman Filter (EKF) and related variants [7,8,9]. However, this structure is highly dependent on the completeness of GNSS solutions, typically requiring at least four visible satellites to perform three-dimensional positioning. When GNSS signals are obstructed or the number of visible satellites is insufficient, the LC structure cannot obtain valid observations, leading to system update interruptions or even positioning failure.

To address the insufficient robustness of the LC architecture in complex environments, the TC structure has been developed. This structure directly incorporates raw GNSS observations—such as pseudorange, Doppler shift, and carrier phase—into the filtering process and jointly estimates them with INS information. Even when fewer than four satellites are visible, it can still provide position or velocity constraints, thereby maintaining system updates and navigation continuity [10]. In recent years, research combining factor graph optimization with carrier phase ambiguity resolution has achieved significant improvements in fusion accuracy and convergence speed, centimeter-level positioning accuracy [11]. However, the TC structure demands stringent system clock synchronization, data interfaces, and model design. Its expanded filter state dimension and significantly increased computational complexity pose higher challenges to real-time performance and embedded resources, limiting its widespread application in low-cost or resource-constrained platforms.

Against this background, various auxiliary constraint strategies have been proposed to enhance the navigation capability of LC structures under degraded GNSS conditions. Zero Velocity Update (ZUPT) is widely applied in pedestrian navigation and stationary vehicle scenarios, constraining velocity to zero when the system is detected to be stationary, effectively suppressing INS error divergence [12]. For wheeled ground vehicles, wheel-odometer measurements can also be incorporated into the integration algorithm to provide forward-speed constraints in the vehicle frame, which help to limit INS error growth during periods of GNSS unavailability or severe signal degradation [13]. Non-Holonomic Constraint (NHC) exploits the characteristic that lateral and vertical velocities of ground vehicles are approximately zero during motion, constructing pseudo-observations to limit error accumulation in specific directions [14]. However, many of these constraint-based methods either rely on additional onboard sensors (such as wheel odometers or other ranging/radar devices) or are tailored to specific motion patterns and vehicle platforms, which may limit their applicability to generic low-cost systems. Additionally, to address the varying motion states and measurement conditions in urban environments, some studies have proposed multi-model Kalman filtering (e.g., MCKN), which processes multiple ZUPT-based models in parallel to enhance system adaptability and robustness [15].

Beyond these constraint methods, recent studies have focused on using Doppler observations to improve the robustness of LC structures [16,17,18]. In scenarios where GNSS signals are severely degraded, such that code pseudorange measurements become too noisy or biased to provide a reliable position fix, Doppler frequency shifts can still be measured with relatively high robustness. In these cases, Doppler observations remain a reliable velocity source and can be used to construct velocity constraints to assist INS estimation, significantly improving continuity and accuracy under weak GNSS conditions [19]. Some studies have also incorporated auxiliary sensor observations, such as Ultra-Wideband (UWB) ranging and millimeter-wave radar, into LC structures to compensate for missing GNSS velocity information, achieving good navigation performance in obstructed environments [20].

In summary, although the LC structure is highly dependent on the completeness of GNSS solutions, its simplicity, low implementation cost, and strong engineering applicability make it valuable in practice. In this study, the main application scenario is low-cost GNSS/INSs for passenger cars and other ground vehicles operating in dense urban environments, where GNSS signals are frequently degraded by blockage and multipath. To balance system simplicity and environmental adaptability, this study proposes an improved LC method that fuses GNSS Doppler observations with IMU attitude information, using only measurements from a low-cost GNSS receiver and an IMU, without introducing additional onboard sensors such as wheel odometers, UWB, or radar. The method aims to enhance positioning continuity and robustness under weak GNSS conditions without significantly increasing system complexity, and to provide a practical and theoretically grounded solution for constructing low-cost, high-reliability integrated navigation systems in challenging environments.

## 2. Doppler–IMU Velocity Estimation

### 2.1. Traditional Doppler Velocity Model

The basic principle of Doppler-based velocity estimation is to directly calculate the platform velocity using the raw Doppler shift observations received by the GNSS receiver. Doppler shift arises from the relative motion between the receiver and the satellite, and its estimate can be directly obtained from the phase change rate output by the Phase-Locked Loop (PLL) [21,22]. Under a first-order approximation, the Doppler shift D of GNSS satellite S relative to receiver R at a given frequency i can be expressed as:

(1) $$D_{R,i}^{S}=f_{R,i}-f_{i}^{S}=\frac{V_{\rho_{R}^{S}}}{c}f_{i}^{S}=\frac{V_{\rho_{R}^{S}}}{\lambda_{i}}$$

where $f_{i}^{S}$ is the frequency of the signal transmitted by the GNSS satellite; $f_{R,i}$ is the frequency of the signal received by the receiver; $V_{\rho_{R}^{S}}$ is the radial velocity of receiver R relative to satellite S; c is the speed of light in vacuum; and $\lambda_{i}$ is the wavelength. When the receiver and the satellite are approaching each other, $D_{R,i}^{S}$ is positive; when the receiver and the satellite are moving apart, $D_{R,i}^{S}$ is negative. Based on the original observation equation, the radial velocity between the receiver and the satellite is expressed as follows:

(2) $$V_{\rho_{R}^{S}}=\lambda_{i}D_{R,i}^{S}={\dot{\rho }}_{R}^{S}+c(d{\dot{t}}_{R}-d{\dot{t}}^{S})+\dot{\mathrm{ion}}+\dot{\mathrm{tro}}+\dot{\varepsilon }$$

where “⋅” is the derivative with respect to time; ${\dot{\rho }}_{R}^{S}$ is the rate of change in the geometric distance between the receiver and satellite; $d{\dot{t}}_{R}$ and $d{\dot{t}}^{S}$ are the receiver and satellite clock drifts, respectively; $i\dot{o}n$ and $t\dot{r}o$ are the ionospheric and tropospheric delay rates, respectively; and $\dot{\varepsilon }$ is the rate of other observation errors, including measurement noise and multipath effects. Most studies consider slowly varying effects, such as ionospheric delay, tropospheric delay, tidal effects, and multipath, to be negligible [23,24,25]. By ignoring these error terms, a single-point, non-differential Doppler observation model is obtained:

(3) $$e_{R}^{S}(V_{R}-V^{S})=\lambda_{i}D_{R,i}^{S}-c(d{\dot{t}}_{R}-d{\dot{t}}^{S})+\dot{\varepsilon }$$

where $e_{R}^{S}$ is a three-dimensional row vector representing the direction cosines of the line connecting the receiver and the satellite; $V_{R}$ and $V^{S}$ are the three-dimensional velocity column vectors of the receiver and satellite in the Earth-Centered Earth-Fixed (ECEF) coordinate system, respectively. The term $e_{R}^{S}(V_{R}-V^{S})$ denotes the inner product between the line-of-sight unit vector $e_{R}^{S}$ and the relative velocity vector $\left(V_{R}-V^{S}\right)$, which gives the projection of the relative velocity onto the line-of-sight direction.

In the conventional GNSS Doppler-based velocity estimation method, to solve for the receiver’s three-dimensional velocity vector $V_{R}=[\begin{array}{ccc} v_{x} & v_{y} & v_{z} \end{array}{]}^{T}$ and the receiver clock drift term $c\cdot d{\dot{t}}_{R}$, Doppler observations from at least four visible GNSS satellites are required [26]. For each satellite $S_{k}$, the corresponding observation equation can be established as follows:

(4) $$e_{R}^{S_{k}}(V_{R}-V^{S_{k}})=\lambda_{i}D_{R,i}^{S_{k}}-c(d{\dot{t}}_{R}-d{\dot{t}}^{S_{k}})+{\dot{\varepsilon }}_{k}$$

expanding $e_{R}^{S_{k}}$ as the unit direction vector from the receiver to satellite, $e_{R}^{S_{k}}=[\begin{array}{ccc} e_{kx} & e_{ky} & e_{kz} \end{array}]$, and denoting the receiver velocity as $V_{R}={\left[\begin{array}{ccc} v_{x} & v_{y} & v_{z} \end{array}\right]}^{T}$, we have:

(5) $$e_{R}^{S_{k}}V_{R}+cd{\dot{t}}_{R}=\lambda_{i}D_{R,i}^{S_{k}}+cd{\dot{t}}^{S_{k}}+e_{R}^{\left(S_{k}\right)}V^{\left(S_{k}\right)}+{\dot{\varepsilon }}_{k}$$

by combining the observation equations from all n visible satellites, a linear observation model in matrix form can be constructed:

(6) $$H\cdot x=v_{D}+\varepsilon$$

where $H=\left[\begin{array}{cc} {\vec{e}}_{1}^{T} & 1\\ {\vec{e}}_{2}^{T} & 1\\ \vdots & \vdots \\ {\vec{e}}_{n}^{T} & 1 \end{array}\right]\in R^{n\times 4}$ is the design matrix, which contains the direction cosines of each satellite and the coefficients of the clock drift term; $x={\left[\begin{array}{cccc} v_{x} & v_{y} & v_{z} & c\cdot d{\dot{t}}_{R} \end{array}\right]}^{T}$ is the state vector to be estimated; $v_{D}=\left[\begin{array}{c} \lambda_{i}D_{R,1}^{S_{1}}+c{d\dot{t}}^{S_{1}}\\ \lambda_{i}D_{R,2}^{S_{2}}+cd{\dot{t}}^{S_{2}}\\ \vdots \\ \lambda_{i}D_{R,n}^{S_{n}}+cd{\dot{t}}^{S_{n}} \end{array}\right]$ is the Doppler observation vector; and $\varepsilon \in R^{n}$ is the observation noise vector.

When n≥4, the receiver’s three-dimensional velocity and clock drift can be estimated using the least squares method as $\hat{x}=(H^{T}H{)}^{-1}H^{T}v_{D}.$

### 2.2. Differential Doppler and IMU Attitude-Aided Velocity Estimation (DDIA-VE)

Although the traditional GNSS Doppler-based velocity estimation method provides high accuracy in open-sky environments, it requires stable signals from at least four satellites to simultaneously solve for the three-dimensional velocity and receiver clock drift. However, in complex environments such as urban canyons, forests, or tunnels, GNSS signals are often blocked or degraded, resulting in insufficient visible satellites. Consequently, the traditional model cannot form a complete set of equations, leading to distorted or even unsolvable velocity estimates.

To overcome this limitation, this paper proposes a low-cost velocity estimation method that integrates the differential Doppler observation model with IMU attitude information. By reducing the dependence on the number of visible satellites, this approach enables robust velocity estimation under low-dimensional observation conditions, thereby maintaining system continuity and robustness in weak GNSS environments.

Before deriving the Differential Doppler and IMU Attitude-Aided Velocity Estimation (DDIA-VE) model, the Non-Differential Doppler and IMU Attitude-Aided Velocity Estimation (NDIA-VE) model is first established to clarify the fundamental principles of combining Doppler observations with IMU attitude information.

#### 2.2.1. Basic Principle of NDIA-VE

To provide a clearer understanding of the fundamental principle of Doppler–IMU fusion for velocity estimation, this section first introduces the case where IMU attitude information is combined with a single satellite’s Doppler observation, assuming that the receiver clock drift is negligible. This process illustrates how the IMU attitude constraint transforms the Doppler-measured radial velocity into the receiver’s overall three-dimensional motion velocity, thereby laying the theoretical foundation for the subsequent development of the differential Doppler model.

**Figure 1:**
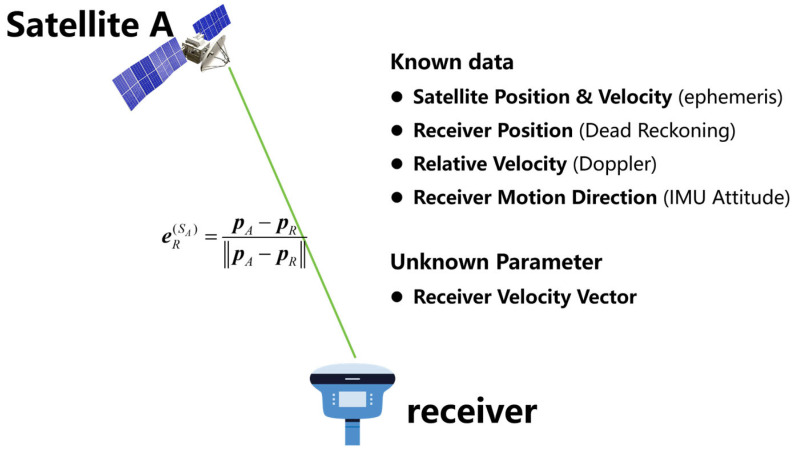
Schematic diagram of the NDIA-VE principle.

As shown in Figure 1, the known data include:

1. The velocity and position of the satellite in the Earth-Centered Earth-Fixed (ECEF) coordinate system, obtained from precise or broadcast ephemerides.
2. The position of the receiver, determined by GNSS absolute positioning or inertial dead reckoning.
3. The Doppler observation values, derived from the observation file, representing the relative line-of-sight velocity between the receiver and the satellite.
4. The motion direction of the receiver in the navigation coordinate system, obtained from IMU attitude updates.

Based on the above information, the receiver’s three-dimensional velocity vector in the Earth-Centered Earth-Fixed (ECEF) coordinate system can be estimated through the following steps:

1. Calculate the Line-of-Sight (LOS) unit vector between the satellite and the receiver (satellite-to-ground direction).
2. Project the satellite velocity onto the LOS vector to obtain the satellite’s velocity component along the LOS direction.
3. Compute the relative velocity between the receiver and the satellite along the LOS direction from the Doppler observation.
4. Derive the receiver’s projected velocity component along the LOS direction.
5. Using the receiver’s motion direction in the navigation coordinate system (provided by the IMU), back-calculate the three-dimensional velocity vector of the receiver in the ECEF coordinate system.

The method discussed in this section estimates the receiver’s velocity by combining single-satellite Doppler observations with IMU attitude information, under the assumption that the receiver clock drift is negligible. However, in practical Doppler-based velocity estimation, the receiver clock drift is a significant error source that cannot be ignored [27,28,29]. To further improve velocity accuracy and effectively eliminate the influence of clock drift, the next subsection introduces the concept of DDIA-VE and describes its computational procedure and implementation in detail.

#### 2.2.2. Basic Principle of DDIA-VE

From Equation (3), it can be seen that the unknown parameters in the non-differential Doppler observation model include the receiver clock drift, the satellite clock drift, and the receiver’s radial velocity along the satellite line of sight. Among these, the satellite clock drift can be obtained from broadcast ephemerides, while the receiver’s radial velocity and clock drift remain as parameters to be estimated. However, under the constrained GNSS observation conditions considered in this study, it is often not possible to solve for the receiver clock drift using conventional parameter estimation methods. Moreover, the influence of satellite clock drift on the velocity estimation results is non-negligible. Therefore, this paper adopts an inter-satellite differencing approach to eliminate the impact of the receiver clock drift in the observation equation, thereby improving the accuracy of velocity estimation.

**Figure 2:**
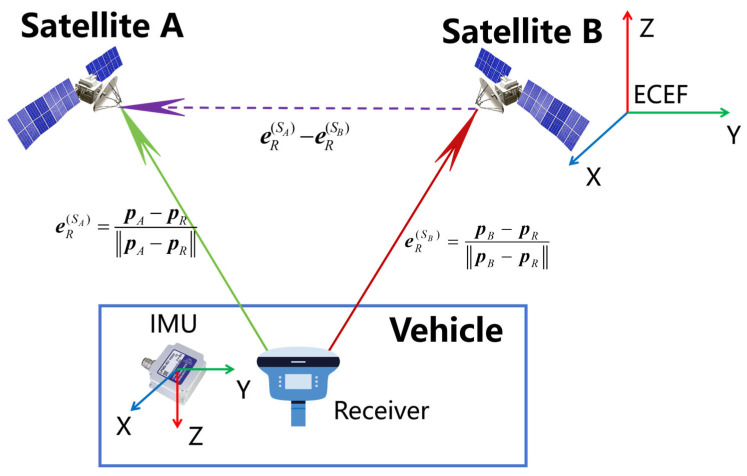
Schematic diagram of the DDIA-VE principle.

As shown in Figure 2, we select two satellites, Satellite A and Satellite B, for observations with the receiver, and write the non-differential Doppler observation equations for the two satellites separately as follows:

For satellites A and B, the non-differential Doppler observation equations can be written as:

(7) $$e_{R}^{S_{A}}(V_{R}-V^{S_{A}})=\lambda_{i}D_{R,i}^{S_{A}}-c(d{\dot{t}}_{R}-{d\dot{t}}^{S_{A}})+{\dot{\varepsilon }}_{A}$$

(8) $$e_{R}^{S_{B}}(V_{R}-V^{S_{B}})=\lambda_{i}D_{R,i}^{S_{B}}-c(d{\dot{t}}_{R}-{d\dot{t}}^{S_{B}})+{\dot{\varepsilon }}_{B}$$

by subtracting Equation (8) from Equation (7), the differential Doppler observation model is obtained:

(9) $$e_{R}^{S_{A}}(V_{R}-V^{S_{A}})-e_{R}^{S_{B}}(V_{R}-V^{S_{B}})=\lambda_{i}(D_{R,i}^{S_{A}}-D_{R,i}^{S_{B}})-c(d{\dot{t}}^{S_{A}}-{d\dot{t}}^{S_{B}})+({\dot{\varepsilon }}_{A}-{\dot{\varepsilon }}_{B})$$

Equation (9) shows that the receiver clock drift term is completely eliminated. It should be emphasized that Equation (9) does not correspond to the conventional GNSS Doppler velocity solution that estimates the three components of the receiver velocity together with the receiver clock drift, which typically requires at least four satellites. Instead, Equation (9) provides a one-dimensional constraint on the projection of the receiver velocity along the satellite-difference vector formed by any pair of satellites, so that only two satellites are sufficient to generate a Doppler-based velocity observation. Since the goal in this section is to obtain the receiver’s three-dimensional velocity under insufficient GNSS observations by fusing the receiver’s motion direction in the navigation frame (provided by IMU attitude data), we next present the detailed procedure for differential Doppler and IMU attitude-aided velocity estimation.

Step 1: Computation of the LOS Unit Vector

Since the equivalent velocity calculated from Doppler measurements is along the satellite-to-receiver line-of-sight direction, it is necessary to first compute the LOS unit vectors. As shown in Figure 2, $e_{R}^{S_{A}}$ and $e_{R}^{S_{B}}$ are the LOS unit vectors from the receiver to satellites A and B, respectively, where

(10) $$e_{R}^{S_{A}}=\frac{P_{A}-P_{R}}{∥P_{A}-P_{R}∥}\;\;e_{R}^{S_{B}}=\frac{P_{B}-P_{R}}{∥P_{B}-P_{R}∥}$$

in Equation (10), $P_{A}$ and $P_{B}$ are the positions of satellites A and B, respectively, which are obtained from satellite ephemerides; $P_{R}$ is the receiver position, derived from GNSS solutions or IMU-based dead reckoning.

Step 2: Projecting the satellite velocity onto the line-of-sight direction

The three-dimensional velocity vectors of the satellites can be obtained from broadcast or precise ephemerides. To derive the receiver velocity along the line-of-sight direction from the Doppler observations, the projection of the satellite velocity onto the line-of-sight must first be computed. Equation (11) gives the line-of-sight velocity components of satellites A and B, denoted as $V_{e}^{S_{A}}$ and $V_{e}^{S_{B}}$, respectively.

(11) $$V_{e}^{S_{A}}=V^{S_{A}}\cdot e_{R}^{S_{A}}\;\;V_{e}^{S_{B}}=V^{S_{B}}\cdot e_{R}^{S_{B}}$$

Step 3: Computing the relative velocity along the line-of-sight from Doppler observations

To convert the Doppler observations into the equivalent velocity along the line-of-sight direction, it should be noted that the calculated results include the receiver clock drift. Equation (12) gives the relative line-of-sight velocities between the receiver and satellites A and B, denoted as $V_{\mathrm{rel}}^{S_{A}}$ and $V_{\mathrm{rel}}^{S_{B}}$, respectively.

(12) $$V_{\mathrm{rel}}^{S_{A}}=\lambda_{i}D_{R,i}^{S_{A}}\;\;V_{\mathrm{rel}}^{S_{B}}=\lambda_{i}D_{R,i}^{S_{B}}$$

Step 4: Differential Line-of-Sight Vector and Differential Projected Velocity

To eliminate the receiver clock drift in Doppler-based velocity estimation, a virtual differential vector $\Delta e_{R}^{S_{AB}}$ is constructed as:

(13) $$\Delta e_{R}^{S_{AB}}=(e_{R}^{S_{A}}-e_{R}^{S_{B}})$$

$\Delta e_{R}^{S_{AB}}$ corresponds to the purple dashed vector $e_{R}^{S_{A}}-e_{R}^{S_{B}}$ shown in Figure 2. Then, the receiver velocity projection along this differential direction, $V_{e}^{S_{AB}}$, is computed. By rearranging Equation (9) and substituting Equation (13), the following equation is obtained, where the left-hand side represents $V_{e}^{S_{AB}}$:

(14) $$\Delta e_{R}^{S_{AB}}V_{R}=\lambda_{i}(D_{R,i}^{S_{A}}-D_{R,i}^{S_{B}})-c(d{\dot{t}}^{S_{A}}-d{\dot{t}}^{S_{B}})+({\dot{\varepsilon }}_{A}-{\dot{\varepsilon }}_{B})+e_{R}^{S_{A}}V^{S_{A}}-e_{R}^{S_{B}}V^{S_{B}}$$

Step 5: Receiver Velocity Direction Unit Vector

According to the non-holonomic constraint assumption, the vehicle has zero lateral and vertical velocities in the body coordinate system, i.e., the velocities along the Y- and Z-axes are zero [30,31,32]. Therefore, the receiver velocity direction unit vector in the body frame is $e_{b}={\left[\begin{array}{ccc} 1 & 0 & 0 \end{array}\right]}^{T}$. It should be noted that this classical NHC is generally valid for wheeled ground vehicles driving on paved roads under normal operating conditions, where lateral tire slip and vertical motion can be neglected. However, in scenarios such as aggressive steering or hard braking, on wet or loose surfaces that cause significant sideslip, or in off-road environments with pronounced vertical motion, this assumption may no longer hold. In such cases, enforcing the non-holonomic constraint may introduce additional modelling errors into the estimated velocity and attitude. A detailed analysis of these situations will be left for future work.

Step 6: Estimation of the Receiver 3D Velocity in ECEF Frame

Since the receiver motion direction in the navigation (NED) frame is known, the rotation matrix from the body frame to the navigation frame is first constructed as:

(15) $$C_{b}^{n}=\left[\begin{array}{ccc} cos\;\psi cos\;\theta & cos\;\psi sin\;\theta sin\;\phi -sin\;\psi cos\;\phi & cos\;\psi sin\;\theta cos\;\phi +sin\;\psi sin\;\phi \\ sin\;\psi cos\;\theta & sin\;\psi sin\;\theta sin\;\phi +cos\;\psi cos\;\phi & sin\;\psi sin\;\theta cos\;\phi -cos\;\psi sin\;\phi \\ -sin\;\theta & cos\;\theta sin\;\phi & cos\;\theta cos\;\phi \end{array}\right]$$

where ψ is the yaw angle, θ is the pitch angle, and ϕ is the roll angle.

Based on the receiver’s geographic position, the rotation matrix from the NED frame to the ECEF frame is given as:

(16) $$C_{n}^{e}=\left[\begin{array}{ccc} -sin\;\varphi cos\;\lambda & -sin\;\lambda & -cos\;\varphi cos\;\lambda \\ -sin\;\varphi sin\;\lambda & \cos\;\lambda & -cos\;\varphi sin\;\lambda \\ \cos\;\varphi & 0 & -sin\;\varphi \end{array}\right]$$

where φ is the latitude and λ is the longitude.

Thus, the overall transformation from the body frame to the ECEF frame is:

(17) $$C_{b}^{e}=C_{n}^{e}C_{b}^{n}$$

Due to installation imperfections, there exists a misalignment error between the IMU and the vehicle coordinate frame, referred to as the misalignment angle error and denoted by $C_{m}^{b}$, where b and m represent the vehicle frame and the IMU frame, respectively. In this paper, $C_{m}^{b}$ is assumed to be constant and is obtained offline from a short straight-line driving calibration of the vehicle. Using the above rotation matrices $C_{b}^{e}$ and taking the misalignment $C_{m}^{b}$ into account, the unit velocity vector of the receiver in the body (IMU) frame, $e_{b}$, can be projected into the ECEF frame and expressed as:

(18) $${\hat{V}}_{\mathrm{ecef}}=C_{b}^{e}{C_{m}^{b}e}_{b}$$

After normalization, the unit velocity direction of the receiver in the ECEF frame is obtained as:

(19) $$e_{\mathrm{ecef}}=\frac{{\hat{V}}_{\mathrm{ecef}}}{∥{\hat{V}}_{\mathrm{ecef}}∥}$$

Finally, combining the differential Doppler–derived projection velocity $V_{e}^{S_{AB}}$ and the differential line-of-sight vector $\Delta e_{R}^{S_{AB}}$, the actual receiver velocity in the ECEF frame can be estimated as:

(20) $$V_{\mathrm{ecef}}=\frac{V_{e}^{S_{AB}}}{e_{\mathrm{ecef}}\cdot \Delta e_{R}^{S_{AB}}}\cdot e_{\mathrm{ecef}}$$

With the above steps, the complete procedure for Doppler–IMU attitude-aided velocity estimation using differential Doppler observations is established. However, it should be noted that when the RMD and the SDV become nearly orthogonal (i.e., their dot product approaches zero), Equation (20) becomes ill-conditioned, making the velocity solution sensitive to Doppler measurement noise and attitude perturbations. Therefore, in the next section we first investigate the geometric singularity that arises when SDV and RMD are nearly orthogonal, analyze the statistical relationship between the velocity error and ∣SDV·RMD∣, and determine an empirical threshold for excluding near-singular epochs. On this basis, we will further focus on analyzing the major factors affecting the velocity estimation accuracy and proposing a velocity data screening strategy under multi-factor constraints.

## 3. Velocity Accuracy Assessment and Multi-Factor Screening Strategy

In GNSS positioning, the Position Dilution of Precision (PDOP) is an important metric used to evaluate the influence of satellite geometric distribution on positioning accuracy. It reflects the extent to which the relative satellite geometry affects the reliability of the navigation solution [33,34,35,36]. Inspired by this concept, in this section, we systematically analyze the factors affecting velocity estimation accuracy from the perspective of satellite elevation and azimuth angles, the geometric relationship between SDV and RMD, as well as SNR of the received satellite signals.

Specifically, satellite elevation and azimuth jointly determine the spatial distribution of the satellite line-of-sight vectors. High-elevation satellites are less likely to be blocked by buildings, have shorter signal paths, and generally suffer from weaker multipath and atmospheric errors; low-elevation satellites, on the other hand, are more easily affected by “urban canyon” effects and reflections from surrounding objects, leading to significantly increased observation noise. The angle between SDV and RMD determines the sensitivity of differential Doppler measurements to the actual motion direction. When ∣SDV·RMD∣ is large, the differential Doppler is highly sensitive to the velocity component along the motion direction, and the noise amplification in the velocity solution is relatively small; when the two are nearly orthogonal, the velocity component in that direction becomes almost unobservable, and the velocity solution becomes extremely sensitive to measurement noise and modeling errors. At the same time, SNR directly reflects the signal strength and noise level at the receiver. When SNR is low or highly fluctuating, the random error of Doppler measurements increases significantly, and the overall velocity accuracy will be limited even if the satellite geometry is favorable.

Based on the above physical analysis, we then use a multi-factor statistical approach to quantitatively evaluate the contributions of SNR, satellite geometry, and the SDV–RMD relationship to the velocity errors in different datasets, and thereby identify the key dominant factors that govern the velocity estimation accuracy.

### 3.1. Experimental Data

In this experiment, three datasets from the GREAT project released in 2024 by the State Key Laboratory of Information Engineering in Surveying, Mapping and Remote Sensing, Wuhan University, are utilized [37]. The datasets provide real-vehicle multi-sensor measurements in complex urban environments, including GNSS observations collected from a Septentrio PolaRx5 receiver operating at 1 Hz and MEMS-IMU measurements provided by an ADIS-16470 unit operating at 100 Hz. A high-precision reference trajectory is obtained by integrating GNSS and a tactical-grade IMU, which is used for accuracy assessment (although the receiver is capable of tracking multiple constellations, only GPS observations are selected for the experiments in this paper).

The key specifications of the low-cost MEMS IMU ADIS-16470 used in this study are summarized in Table 1, including a sampling rate of 100 Hz, a typical gyroscope bias of 8 °/h, an accelerometer bias of 13 mGal, an angle random walk of 0.34 °/$\sqrt{h}$, and a velocity random walk of 0.037 m/s/√$\sqrt{h}$ [38].

### 3.2. Geometric Singularity Analysis Based on SDV–RMD Alignment

**Figure 3:**
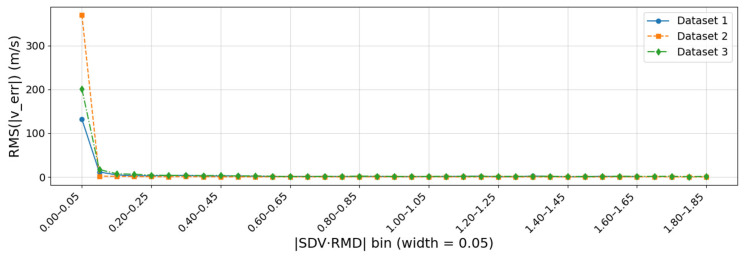
Relationship between ∣SDV·RMD∣ and RMS Doppler-based velocity error for three urban driving datasets (bin width = 0.05).

To quantitatively evaluate the impact of the geometric relationship between SDV and RMD on the velocity estimation accuracy, this study adopts the scalar metric ∣SDV·RMD∣ to characterize their alignment. For each dataset, the magnitude of the Doppler-based velocity error $|v_{\mathrm{err}}|$ is grouped according to ∣SDV·RMD∣ (with a bin width of 0.05), and the RMS velocity error is computed within each bin. The results are shown in Figure 3.

As can be seen from Figure 3, when ∣SDV·RMD∣ <0.05, the velocity errors in the corresponding bins of all datasets are much larger than those in neighboring bins, indicating that the scale-factor equation is severely ill-conditioned in this region and that even small Doppler measurement noise and attitude perturbations are greatly amplified in the velocity solution. Once ∣SDV·RMD∣ exceeds 0.05, the RMS velocity error rapidly decreases and remains at a relatively low and stable level over a wide range of values. These results show that configurations where SDV and RMD are nearly orthogonal constitute the main source of geometric singularity.

Therefore, in the subsequent analyses ∣SDV·RMD∣ =0.05 is adopted as a practical threshold for avoiding singular configurations. Epochs with ∣SDV·RMD∣ <0.05 are directly discarded and excluded from the Doppler-based velocity estimation and subsequent statistical analysis, while only samples satisfying ∣SDV·RMD∣ >0.05 are retained for the multi-factor influence analysis in Section 3.3 and for the design of the data screening strategy.

The proportion of epochs removed by this singularity-avoidance rule remains relatively small for all three datasets. Table 2 summarizes the total number of available samples and the number and percentage of epochs discarded due to ∣SDV·RMD∣ <0.05, as well as the proportion of samples retained for subsequent analysis.

Here, $N_{\mathrm{all}}$ denotes the total number of samples with valid SNR and ∣SDV·RMD∣, $N_{<0.05}$ is the number of epochs with ∣SDV·RMD∣ <0.05, and $N_{\mathrm{remain}}$ is the percentage of samples retained after applying the singularity-avoidance threshold.

### 3.3. Multi-Factor Influence Analysis on Differential Doppler Velocity Accuracy

To facilitate the subsequent evaluation of velocity availability in integrated navigation and the optimization of differential combinations under multi-satellite conditions, this section systematically analyzes the factors affecting differential Doppler velocity estimation accuracy using real-world data. The analysis procedure includes data preprocessing and gross-error removal, correlation analysis, fixed-threshold validation, and the development of a multi-factor screening strategy. Through these steps, the influence patterns and mechanisms of various geometric factors on velocity errors are identified and quantified, enabling the determination of the dominant factors affecting velocity accuracy. This provides a quantitative basis for subsequent data availability assessment and differential combination strategies.

#### 3.3.1. Data Preprocessing and Gross-Error Rejection

Following the steps in Section 2.2, the Doppler differential velocity for all possible satellite combinations is computed at each epoch. To quantify the velocity accuracy, the velocity error vector is defined as

(21) $$\Delta v=V_{ecef}-V_{ecef}^{true}$$

where $V_{ecef}={\left[\begin{array}{ccc} v_{x} & v_{y} & v_{z} \end{array}\right]}^{\;T}$ is the receiver’s three-dimensional velocity in the ECEF frame obtained from differential Doppler velocimetry, and $V_{ecef}^{true}={\left[\begin{array}{ccc} v_{x,true} & v_{y,true} & v_{z,true} \end{array}\right]}^{\;T}$ is the ground-truth ECEF velocity.

The Euclidean norm of Δv is used to characterize the magnitude of the velocity error:

(22) $$|err_{v}|\;=\sqrt{(v_{x}-v_{x,true}{)}^{2}+(v_{y}-v_{y,true}{)}^{2}+(v_{z}-v_{z,true}{)}^{2}}$$

After applying the singularity-avoidance threshold described in Section 3.2, the velocity errors of the three datasets are statistically analyzed, and the corresponding standard deviations are summarized in Table 3. To ensure the reliability of the subsequent correlation analysis, the outlier rejection threshold for each dataset is set to three times the RMS velocity error [39,40]; when the velocity-error norm at a given epoch exceeds this threshold, the corresponding observation is regarded as an outlier and removed.

Here, N denotes the number of samples after singularity-avoidance filtering, RMS is the root-mean-square of the velocity-error norm, 3RMS is three times this RMS value and is used as the outlier rejection threshold, $N_{\mathrm{out}}$ is the number of samples rejected as outliers, and $N_{\mathrm{remain}}$ is the percentage of samples retained after outlier removal.

#### 3.3.2. Impact of Attitude Errors on Differential Doppler Velocity Estimation

Before analyzing the various factors that affect the accuracy of differential Doppler velocity estimation, it is first necessary to clarify whether attitude errors from a low-cost IMU will significantly impact the proposed method when the GNSS is unable to provide positioning information. In such cases, the system must rely solely on the IMU for autonomous inertial navigation, and attitude drift caused by gyroscope biases and other errors may degrade the velocity estimation performance.

**Figure 4:**
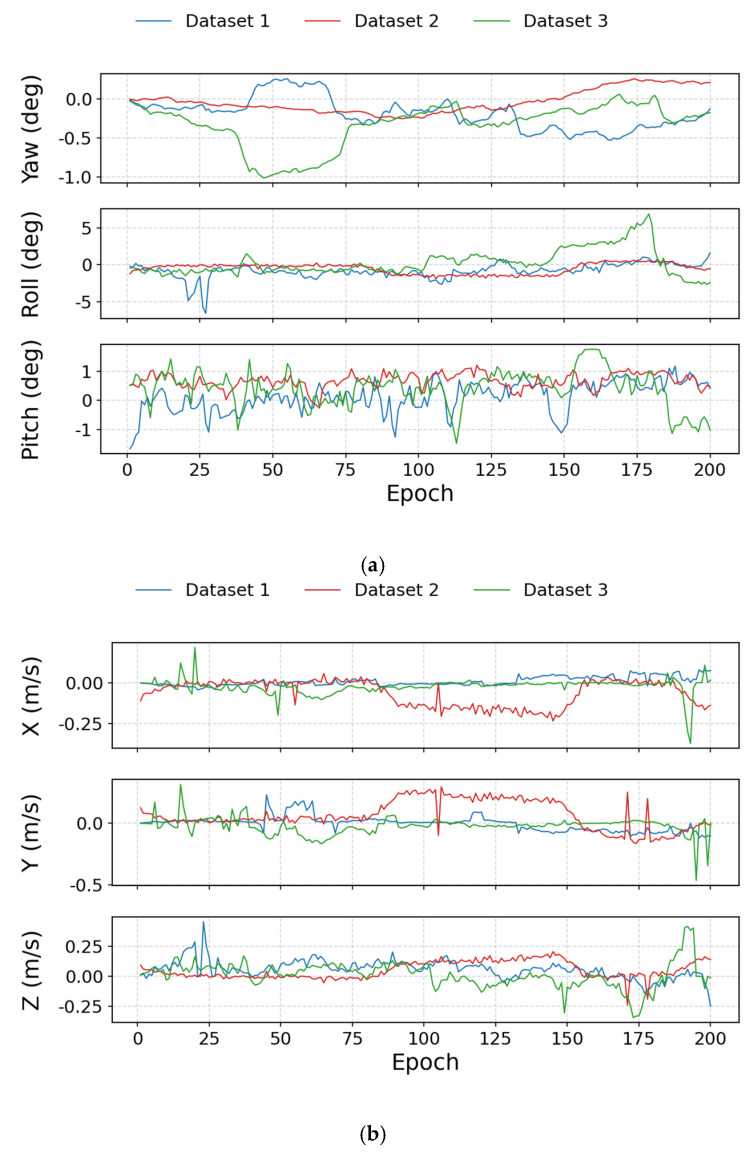
(**a**) Attitude Error Curves within 200 s; (**b**) Velocity Difference Curves within 200 s.

To quantitatively evaluate this effect, the first 200 epochs of each dataset are selected to simulate a GNSS signal outage. For each dataset, two inter-satellite differential Doppler velocity solutions are computed: (1) using the high-precision attitude obtained from the fusion of GNSS and a tactical-grade IMU as the reference attitude; and (2) using the attitude obtained from inertial navigation with the low-cost IMU (ADIS-16470). The difference between the two velocity solutions reflects the additional Doppler-based velocity error introduced by IMU attitude errors. At the same time, the attitude errors of the IMU solution with respect to the reference attitude over the same interval are statistically analyzed. The corresponding results are presented in Figure 4 and Table 4.

The statistics show that, for all three datasets, the RMS errors of yaw, roll, and pitch remain within the sub-degree to 1–2° range (yaw: approximately 0.15–0.43°, roll: 0.87–1.81°, pitch: 0.54–0.75°). Under this level of attitude accuracy, the RMS of the differences between the differential Doppler velocities computed from the reference attitude and those computed from the IMU attitude are 0.028–0.100 m/s in the X direction, 0.060–0.135 m/s in the Y direction, and 0.087–0.110 m/s in the Z direction. Compared with the overall velocity RMS obtained under weak-GNSS conditions in this paper (Table 3), the additional velocity error caused by attitude errors is clearly smaller and plays a secondary role.

Therefore, for the low-cost IMU used in this study and GNSS outage durations on the order of 200 s, attitude drift does not become the dominant error source in differential Doppler velocity estimation; its impact is limited and remains within an acceptable range. This conclusion provides the basis for the subsequent multi-factor analysis, allowing us to focus on other factors such as signal-to-noise ratio (SNR), elevation angle, and satellite geometry.

#### 3.3.3. Correlation Analysis

This study employs both Pearson and Spearman correlation coefficients to assess the statistical relationships between geometric parameters and velocity errors. The Pearson coefficient, which reflects linear correlation between variables [41], is defined as

(23) $$r_{xy}=\frac{\sum_{i=1}^{n}\left(x_{i}-\bar{x})(y_{i}-\bar{y}\right)}{\sqrt{\sum_{i=1}^{n}(x_{i}-\bar{x}{)}^{2}\;}\sqrt{\sum_{i=1}^{n}(y_{i}-\bar{y}{)}^{2}}}$$

In this expression, $r_{xy}$ is the correlation coefficient between the error-influencing factor x and the norm of the differential-Doppler velocity error y. The value of $r_{xy}$ lies within the range [−1, 1]. $x_{i}$ and $y_{i}$ denote the values of the i-th valid sample (after gross-error removal); $\bar{x}$ and $\bar{y}$ are the corresponding sample means; and n is the total number of valid samples.

Because the Pearson coefficient is sensitive only to linear dependence, the correlation of some variables may be underestimated when a nonlinear but monotonic relationship exists. To address this limitation, the Spearman rank correlation coefficient is further adopted, which measures monotonic association by comparing rank differences while preserving correlation directionality [42]:

(24) $$\rho_{xy}=1-\frac{6\sum_{i=1}^{n}d_{i}^{\;2}}{n(n^{2}-1)}$$

Here, $\rho_{xy}$ is the rank correlation coefficient used to measure the strength of the monotonic relationship between the error-influencing factor x and the norm of the differential-Doppler velocity error y. The value of $\rho_{xy}$ lies within the range [−1, 1]. $d_{i}=R(x_{i})-R(y_{i})$ denotes the rank difference in the i-th sample between variables x and y.

**Figure 5:**
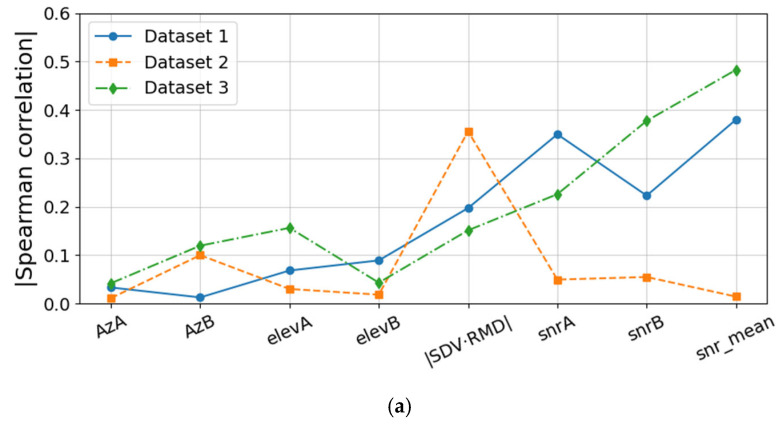

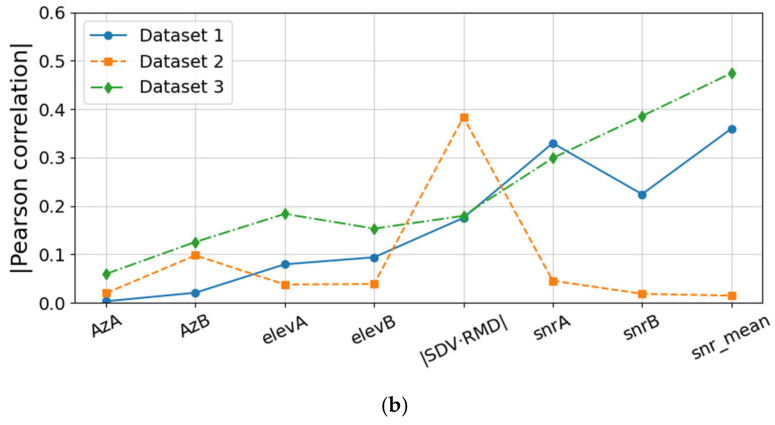
(**a**) Absolute Pearson correlation coefficients between influencing factors and velocity error; (**b**) Absolute Spearman correlation coefficients between influencing factors and velocity error.

To analyze the influence of multiple factors on differential Doppler velocity errors, correlation computation and comparison are performed on three sets of GREAT real-world datasets. After outlier removal, the sample sizes for the three datasets are 10,343, 49,309, and 11,024, respectively. For each dataset, Pearson and Spearman correlations are computed after outlier removal to characterize linear and monotonic relationships, respectively. The results are shown in Figure 5.

In Figure 5, the horizontal axis variables AzA, AzB, elevA, elevB, ∣SDV·RMD∣, snrA, snrB, and snr_mean represent the azimuth angles and elevation angles of satellites A and B, the absolute value of the geometric relationship between SDV and RMD, SNRs of satellites A and B, and the average SNR, respectively. From the results in Figure 5, it can be observed that in Datasets 1 and 3, the average SNR is the primary factor affecting the accuracy of differential Doppler velocity estimation, while in Dataset 2, the geometric relationship between SDV and RMD becomes the dominant factor. Considering differences in observation environments and dynamic changes, we further analyzed the mean SNR and the stability of the mean SNR of differential satellite observations. The results are shown in Figure 5.

**Figure 6:**
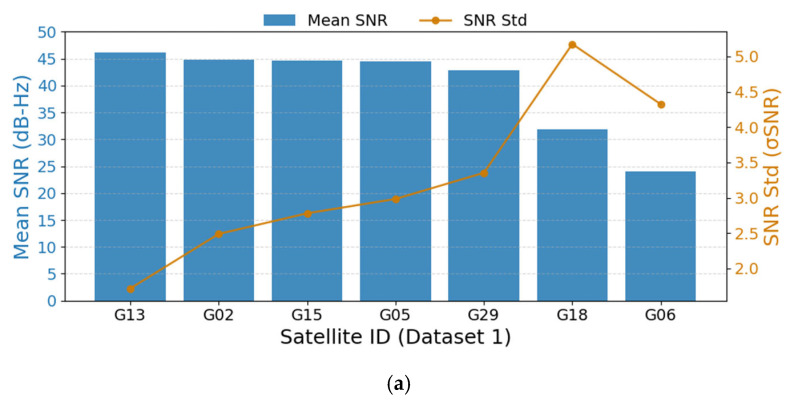

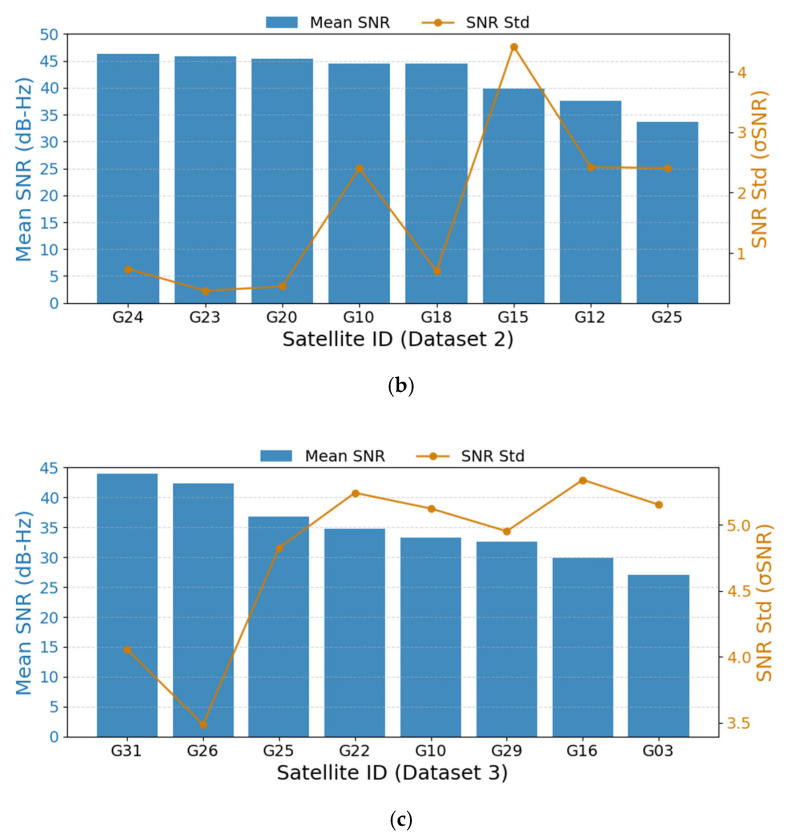
(**a**–**c**), respectively, present the mean SNR and the stability of the mean SNR for the three datasets.

In Figure 6, the blue bar represents the average of the mean SNR values for all differential combinations that include the corresponding satellite, and the orange dots indicate the standard deviation of these mean SNR values. Table 5 presents the average of the mean SNR values and the standard deviation of these mean values for all differential satellite vectors.

According to Figure 6 and Table 5, the mean SNR of Dataset 2 is 42.47 dB-Hz, and the standard deviation of SNR over the entire period is 3.15 dB-Hz, indicating that this dataset was collected under favorable observation conditions and in a relatively stable surrounding environment. Overall, Dataset 2 has the best observation conditions among the three datasets.

For Dataset 1, the mean SNR is also 42.47 dB-Hz, which is relatively high; however, its standard deviation reaches 5.37 dB-Hz, implying weaker signal stability and larger variations in the observation environment. In contrast, Dataset 3 has the lowest mean SNR, only 38.26 dB-Hz, with a standard deviation of 5.32 dB-Hz, indicating that its acquisition environment is relatively poor.

In combination with the correlation results shown in Figure 5, the following conclusions can be drawn:

When the SNR is sufficiently high and stable, the geometric relationship between SDV and RMD becomes the dominant factor affecting the accuracy of differential Doppler velocity estimation. Conversely, when the SNR is relatively low or unstable, SNR becomes the primary factor influencing the velocity estimation accuracy.

#### 3.3.4. Fixed-Threshold Validation and Development of Multi-Factor Filtering Strategy

To evaluate the influence of SNR and spatial geometric factors on Doppler-based velocity accuracy, this study adopts a Fixed-Threshold Method. First, different SNR thresholds are applied to analyze the effect of the spatial geometric factor ∣SDV·RMD∣ on velocity accuracy within various SNR intervals. Subsequently, the velocity measurement accuracy under different combinations of SNR and ∣SDV·RMD∣ thresholds is statistically examined. This procedure provides a quantitative basis for data screening and selection in subsequent Doppler velocity constraint algorithms.

**Figure 7:**
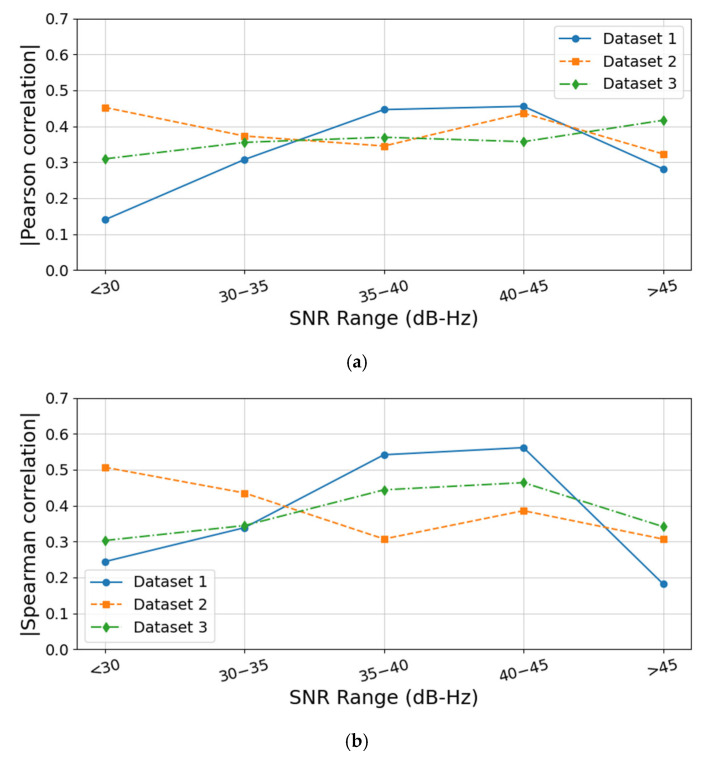
(**a**) Pearson correlation of spatial geometric factor under different SNR ranges; (**b**) Spearman correlation of spatial geometric factor under different SNR ranges.

Figure 7 illustrates the correlation coefficients between the spatial geometric factor and differential Doppler velocity accuracy across three experimental datasets under different SNR ranges. As shown in Figure 7, for the first and third datasets, the influence of the spatial geometric factor on velocity accuracy is relatively weak when the SNR is below 35, but becomes more significant once the SNR exceeds 35. In contrast, for the second dataset—characterized by higher and more stable SNR—the influence does not exhibit an obvious monotonic trend across different SNR levels; however, the correlation coefficient remains consistently high, indicating that the geometric factor remains a non-negligible contributor to velocity accuracy.

These results further demonstrate that when the SNR is sufficiently high and signals are stable, the spatial geometric factor becomes a dominant influence on differential Doppler velocity accuracy. Conversely, in scenarios with low or unstable SNR, its effect becomes overshadowed by the impact of SNR.

**Figure 8:**
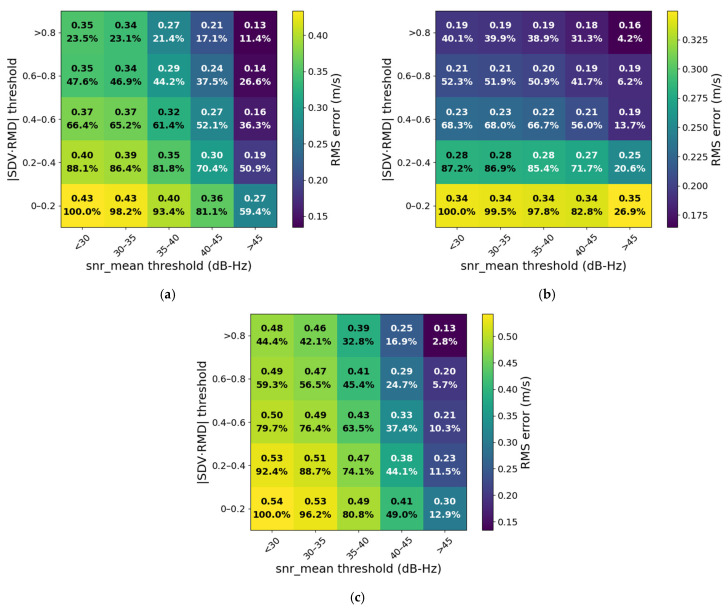
Heatmap of velocity error and data retention rate under different SNR and spatial geometric factor thresholds for the three datasets: (**a**) Dataset 1, (**b**) Dataset 2, and (**c**) Dataset 3.

To provide quantitative guidance for the selection and screening of Doppler-based velocity measurements in subsequent velocity constraint algorithms, this study performs a statistical analysis of the RMS velocity error and data retention rate under joint thresholding of SNR and the geometric factor ∣SDV·RMD∣. Corresponding threshold heatmaps are illustrated in Figure 8.

In Figure 8, the numbers centered within each colored block represent the RMS velocity error and the corresponding data retention rate under the given threshold conditions. The results show that the overall RMS decreases significantly as the thresholds on both SNR and ∣SDV·RMD∣ increase, indicating that higher signal quality and favorable geometric conditions contribute to improved Doppler-based velocity estimation accuracy. However, the data retention rate decreases accordingly. Across the three datasets, when SNR exceeds 45, all achieve a velocity accuracy of approximately 0.3 m/s, while the retention rates of the second and third datasets drop below 30%, with only 12.9% remaining in the latter. Conversely, when SNR > 40 and ∣SDV·RMD∣ >0.2, the first and second datasets retain more than 70% of the data, and the third maintains approximately 44%, enabling satisfactory velocity accuracy while preserving higher data availability. It should be emphasized that these values should be regarded as empirically tuned thresholds for complex urban environments rather than universal constants.

Therefore, in practical applications, an appropriate trade-off between accuracy and availability must be made in accordance with specific mission requirements. The threshold heatmap can thus serve as an important reference for observation data screening and weighting strategy design in subsequent research on Doppler-based velocity constraint algorithms.

## 4. Discussion

This study proposes a differential Doppler and IMU attitude-aided velocity estimation method for GNSS-challenged conditions in complex urban environments. Here, “complex urban environments” refer to urban road networks characterized by densely distributed buildings, frequent partial or intermittent GNSS signal blockages, complex road geometries (such as overpasses and intersections), and relatively diverse vehicle dynamics. Experimental analyses on three sets of real vehicle datasets demonstrated that the proposed method enables reliable 3D velocity estimation even when only two satellites are available, overcoming the inherent limitations of traditional GNSS Doppler-based algorithms which require at least four satellites.

The experimental results indicate that the dominant factors influencing velocity accuracy exhibit strong environmental dependence. Specifically, when satellite signal SNR is relatively low or fluctuating, Doppler measurement noise becomes the primary contributor to velocity degradation. In contrast, under conditions of high and stable SNR, the spatial geometric relationship between the differential line-of-sight vectors and the receiver’s motion direction plays a more critical role in determining velocity estimation accuracy.

On this basis, this paper proposes a velocity measurement data usability evaluation and selection strategy based on SNR and spatial geometric relationship indicators. The heatmap analysis demonstrated that applying threshold-based screening can effectively improve velocity accuracy while maintaining acceptable availability. The results not only validate the effectiveness of the proposed fusion-based velocity estimation method but also provide quantitative and practical data-screening and quality-control criteria for incorporating Doppler velocity into GNSS/INS integrated navigation systems.

Nevertheless, several limitations should be acknowledged. First, the proposed method relies on accurate IMU attitude information, and inertial drift during long GNSS outages may lead to performance degradation. Second, when fewer than two satellites are visible, it is no longer possible to form an effective inter-satellite differential combination; under the influence of receiver clock drift, a single satellite is insufficient to provide reliable radial velocity measurements, and the proposed method will also fail in this case. In addition, this study only considers low-cost MEMS IMU and single-frequency GNSS data; multi-frequency observations, PPP augmentation, or integration with visual/laser odometry are expected to further enhance system robustness in more challenging environments. Future research will focus on (1) extending the fusion framework into EKF-based tight coupling, (2) leveraging multi-sensor redundancy, and (3) developing adaptive weighting strategies for differential Doppler combinations in dynamic urban scenes.

## 5. Conclusions

This paper presents a low-cost Doppler and IMU attitude-aided velocity estimation solution suitable for GNSS-limited environments. By eliminating receiver clock drift via inter-satellite differencing and introducing motion direction constraints from IMU, the method significantly improves velocity observability under reduced satellite visibility. Experiments conducted on complex urban road scenarios confirm that:

1. The proposed method enables continuous and robust 3D velocity estimation with as few as two satellites.
2. A dual-indicator screening approach based on SNR and differential geometric features effectively enhances Doppler velocity accuracy and availability.

The proposed technique is well-suited for low-cost autonomous driving, unmanned ground vehicles, and pedestrian navigation operating in GNSS-challenged environments. Future work will further integrate this technology into complete multi-sensor navigation systems to support reliable positioning in more diverse and complex scenarios.

## Figures and Tables

**Table 1:** Key specifications of the MEMS IMU used in the experiments.

Model	Parameter	Gyro	Accel
ADIS-16470	Bias	8 °/h	0.34 °/√h
	Random walk	$0.34\;{}^{\circ}/\sqrt{h}$	$0.037\;m/s/\sqrt{h}$

**Table 2:** Statistics of samples removed by the ∣SDV·RMD∣ <0.05  singularity-avoidance rule.

	$N_{\mathrm{all}}$	$N_{<0.05}$	$N_{\mathrm{remain}}$
Dataset 1	11,035	514	95.34%
Dataset 2	53,317	3449	93.53%
Dataset 3	11,590	387	96.66%

**Table 3:** RMS velocity error and 3RMS-based outlier removal statistics for three datasets.

	N	RMS (m/s)	3RMS (m/s)	$N_{\mathrm{out}}$	$N_{\mathrm{remain}}$
Dataset 1	10,521	2.92	8.76	178	10,343 (98.3%)
Dataset 2	49,868	0.66	1.99	559	49,309 (98.9%)
Dataset 3	11,203	4.15	12.45	178	11,024 (98.4%)

**Table 4:** Attitude RMS errors and the corresponding Doppler–IMU velocity differences.

Att/Vel		Dataset 1	Dataset 2	Dataset 3
Attitude	Yaw (deg)	0.274	0.153	0.433
	Roll (deg)	1.305	0.870	1.812
	Pitch (deg)	0.540	0.718	0.747
Velocity	x (m/s)	0.028	0.100	0.054
	y (m/s)	0.060	0.135	0.074
	z (m/s)	0.098	0.087	0.110

**Table 5:** The mean SNR and the stability of the mean SNR for the three datasets.

	Mean SNR (dB-Hz)	Std of SNR (dB-Hz)
Dataset 1	42.16	5.37
Dataset 2	42.47	3.15
Dataset 3	38.26	5.32

## Data Availability

The experimental data used in this paper come from the GREAT (GNSS+ Research, Application and Teaching) dataset, released by the research team of the School of Geodesy and Geomatics at Wuhan University in November 2024. This dataset was collected in typical complex urban road network environments and provides raw observations from multiple onboard sensors, including GNSS and IMU. The dataset is publicly available and can be downloaded from: https://github.com/GREAT-WHU/GREAT-Dataset.git (accessed on 2 November 2025).
